# Respond of the different human cranial bones to pin-type head fixation device

**DOI:** 10.1007/s00701-021-04728-z

**Published:** 2021-01-29

**Authors:** Alissa Visentin, Kristina van Dooren, Jan Mertens, Olivier Brina, Karl Schaller

**Affiliations:** 1 Department of Neurosurgery, University of Geneva Medical Center & Faculty of Medicine, University of Geneva, Swiss Foundation for Innovation & Training in Surgery (SFITS), Rue Gabrielle-Perret-Gentil 4, Geneva, Switzerland; 2 Black Forest Medical Group, Freiburg, Germany; 3 Division of Neuroradiology, University of Geneva Medical Center & Faculty of Medicine, University of Geneva, Geneva, Switzerland

**Keywords:** Bone response, Fresh-frozen human specimen, Neurosurgery, Skull clamp, Pin-type head fixation device

## Abstract

**Background:**

At this juncture, there is no consensus in the literature for the use and the safety of pin-type head holders in cranial procedures.

**Methods:**

The present analysis of the bone response to the fixation of the instrument provides data to understand its impact on the entire skull as well as associated complications. An experimental study was conducted on fresh-frozen human specimens to analyze the puncture hole due to the fixation of each single pin of the pin-type head holder. Cone-beam CT images were acquired to measure the diameter of the puncture hole caused by the instrument according to several parameters: the pin angle, the clamping force, and different neurosurgical approaches most clinically used.

**Results:**

The deepest hole, 2.67 ± 0.27 mm, was recorded for a 35° angle and a clamping force of 270 N at the middle fossa approach. The shallowest hole was 0.62 ± 0.22 mm for the 43° angle with a pinning force of 180 N in the pterional approach. The pterional approach had a significantly different effect on the depth of the puncture hole compared with the middle fossa craniotomy for 270 N pinning at 35° angle. The puncture hole measured with the 43° angle and 180 N force in prone position is significantly different from the other approaches with the same force.

**Conclusions:**

These results could lead to recommendations about the use of the head holder depending on the patient’s history and cranial thickness to reduce complications associated with the pin-type head holder during clinical applications.

## Introduction

**Fig. 1:**
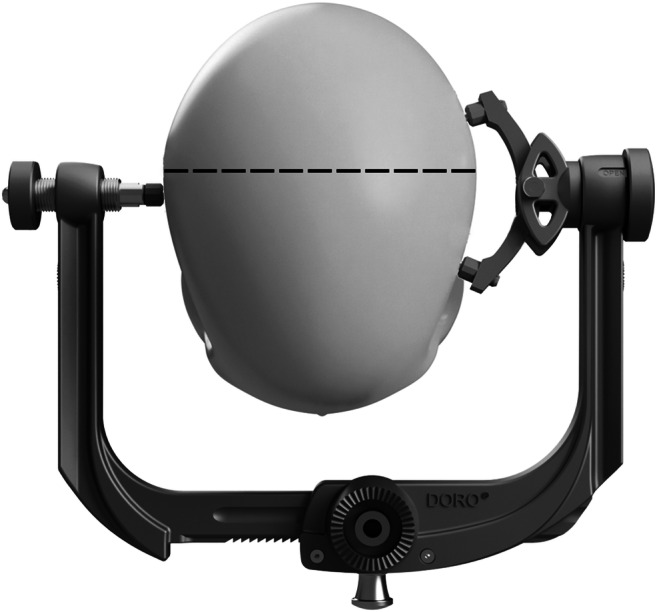
Model created and rendered with SOLIDWORKS Premium 2019 SP4.0; modification and converting into *eps with Adobe Photoshop 22.0

The pin-type head fixation device (HFD) is a surgical instrument that allows the immobilization of a patient’s head in a determined and safe position during neurosurgical procedures [22]. The commonly used type deploys three pins, on one-side one and contralateral two [4]. The single pin is combined with a force gauge screw that adjusts the force applied to the skull. To prevent an unintended movement of the patient’s head during the operation, it is essential that the HFD is correctly positioned and that an adequate force is applied. The placement of the HFD to the equator of the patient’s head and placing two of the three pins below it is an important concept to ensure patient safety during neurosurgical operations. The risk of head slippage is reduced when the instrument is placed at the equator and the patient’s head “falling into the pins” due to gravity. If the HFD is placed too high, the risk of slipping is increased because the pins can no longer support the weight of the head [14, 20] (Fig. 1; front view of a representation of the patient in prone position during a neurosurgical operation). The HFD is fixed as close as possible to the equator of the skull illustrated by the dotted line with two of three pins below the same). HFD also helps to ensure accuracy during neuronavigation [4, 6, 16]. In literature, recommended forces are 270–360 N for adults, but fragile bone regions and neurovascular structures should be avoided [4, 8, 14, 20]. For the pediatric population, the use of HFD is recommended for patients > 3 years [4, 20]. There are widely accepted doctrines as well as recommendations for the application of HFD in clinic [4, 14, 20]. Yet, there is no scientifically determined upper limit for pinning forces utilized among adults that are tolerated by the human skull. In addition, there is still disagreement about the appropriate force to be applied in children [3, 14].

The most common complications reported in the literature from head support are skull fractures, epidural hematomas, air embolisms, and unintended head movement during surgery [4, 8–10, 18]. Due to the design of common HFD, skull fractures predominantly occur on the single-pin side if pinning force is escalated whereas the displacement of pins occurs on the 2-pin side if pinning force is insufficient. These accidents are multifactorial, including failure of the contact mechanics, the pinning technique, and the patient’s history [23]. In 2016, the US Food and Drug Administration (FDA) circulated a safety communication pointing out the scientific neglect of complications leading to the skull and facial injuries [18]. However, complications remain rare events if the patient is not known to have a history of cranial pathology [4, 8].

For children, incidents are more frequent due to a thinner cranial bone thickness, fontanelles, and mobile structures [3, 8, 19, 21]. There is more literature available on complications among the pediatric population compared with the adult population but remains underestimated as few surgeons report their experience in the literature [4].

Despite the daily use of the HFD in the operating room, there are no evidence-based guidelines for its use, especially for adequate pinning forces to avoid complications during its utilization. The goal of this research was to study the cranial responds to the HFD. It experimentally determined the response of the bone to the pin of the single-pin side of the HFD depending on three factors, the pin angle, applied force, and the different pin placing locations.

## Materials and methods

### Composition of the study

The study was conducted between October 2018 and December 2019 in Geneva. Four approaches, applied in clinical practice, were used as reference for pin placing: the bifrontal, pterional, and middle fossa approach and the prone position according to Whitney et al. [20]. Each treatment combination of the three factors pin angle, force, and approach was repeated three times (Table 1).

**Table 1:** Combinations of treatments utilized in the experiment

Treatment	Pin angle (°)	Force (N)	Approach
1	43	180	Bifrontal
2	43	180	Pterional
3	43	180	Middle fossa
4	43	180	Prone/ventral position
5	43	270	Bifrontal
6	43	270	Pterional
7	43	270	Middle fossa
8	43	270	Prone/ventral position
9	43	360	Bifrontal
10	43	360	Pterional
11	43	360	Middle fossa
12	43	360	Prone/ventral position
13	35	180	Bifrontal
14	35	180	Pterional
15	35	180	Middle fossa
16	35	180	Prone/ventral position
17	35	270	Bifrontal
18	35	270	Pterional
19	35	270	Middle Fossa
20	35	270	Prone/ventral position
21	35	360	Bifrontal
22	35	360	Pterional
23	35	360	Middle fossa
24	35	360	Prone/ventral position

### Description of the equipment

Nine adult defrosted fresh-frozen human specimens were utilized. Specimens with cranial pathology visible on the cone-beam CT (CBCT) or previously utilized in medical training activities were excluded. The inclusion of specimens was not done according to age, sex, or ethnicity.

**Fig. 2:**
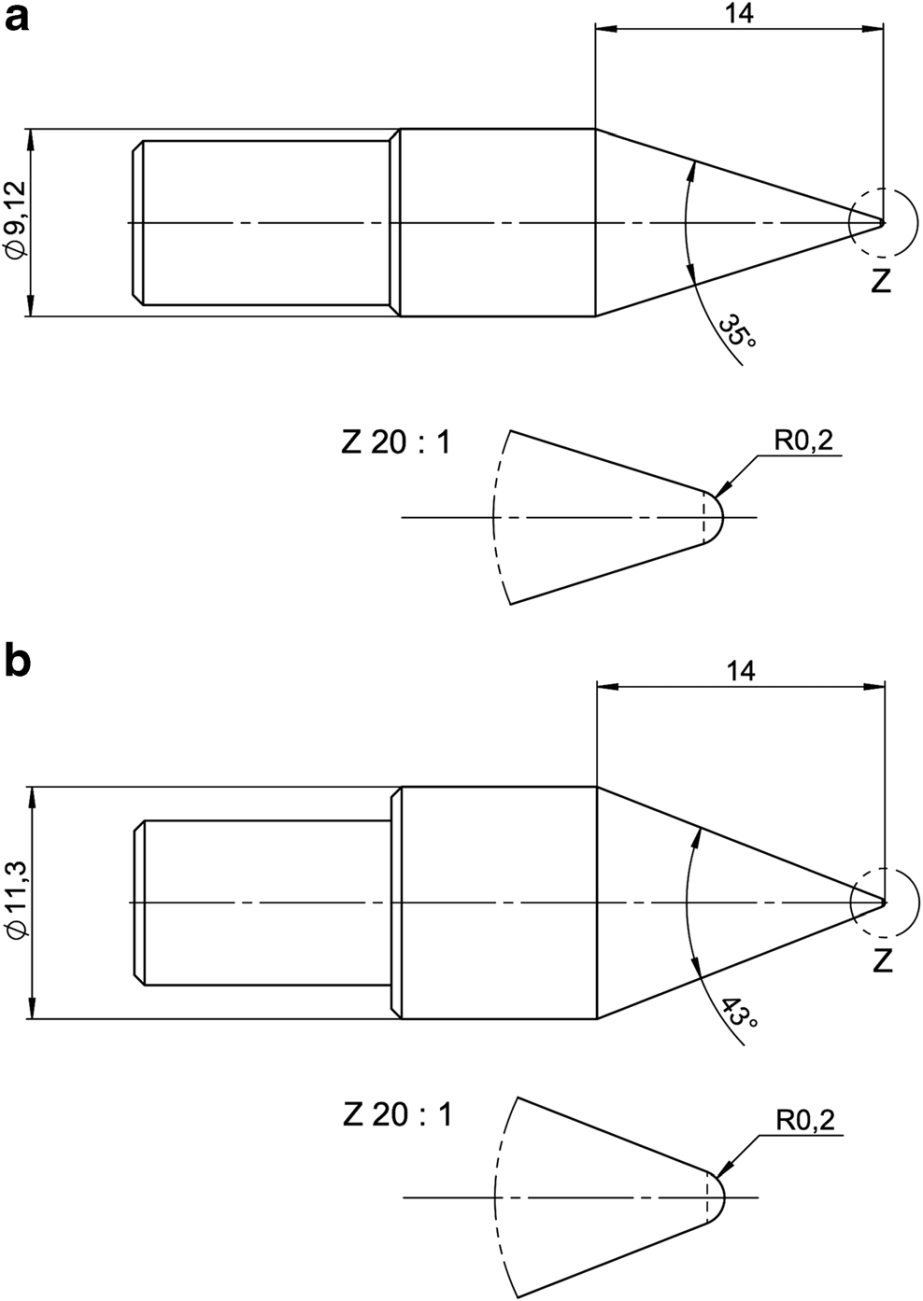
RStudio Team (2016). RStudio: Integrated Development for R. RStudio, Inc., Boston, MA, URL: http://www.rstudio.com

**Fig. 3:**
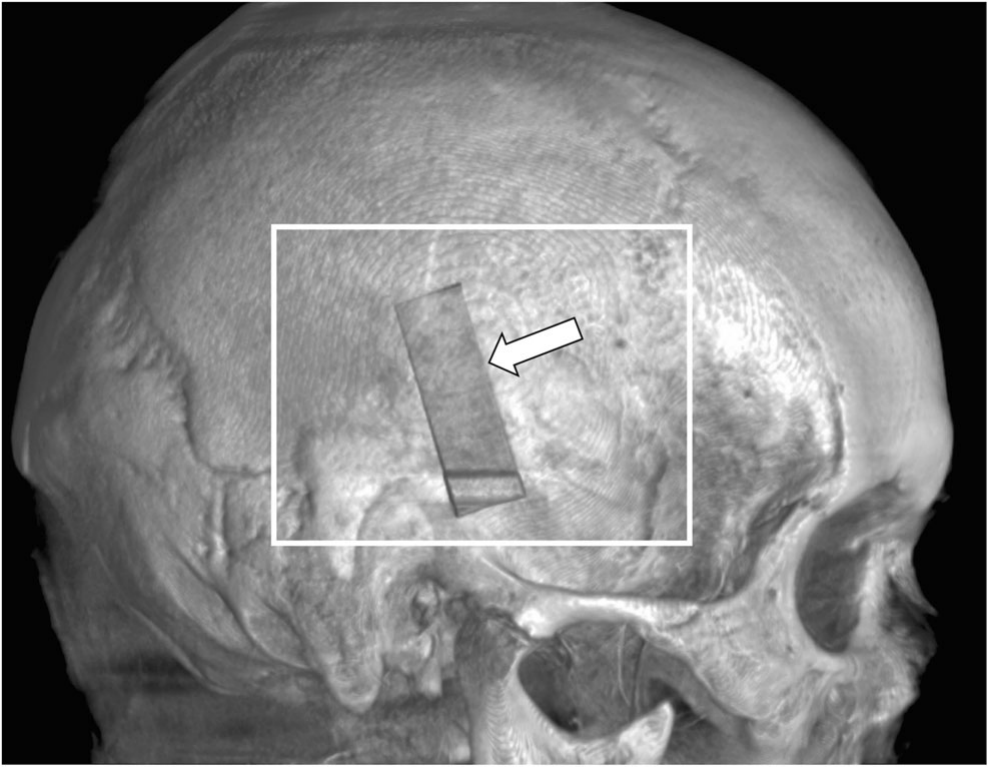
Artis Zeego workstation (Syngo XWP VD 11B) in the DICOM

**Fig. 4:**
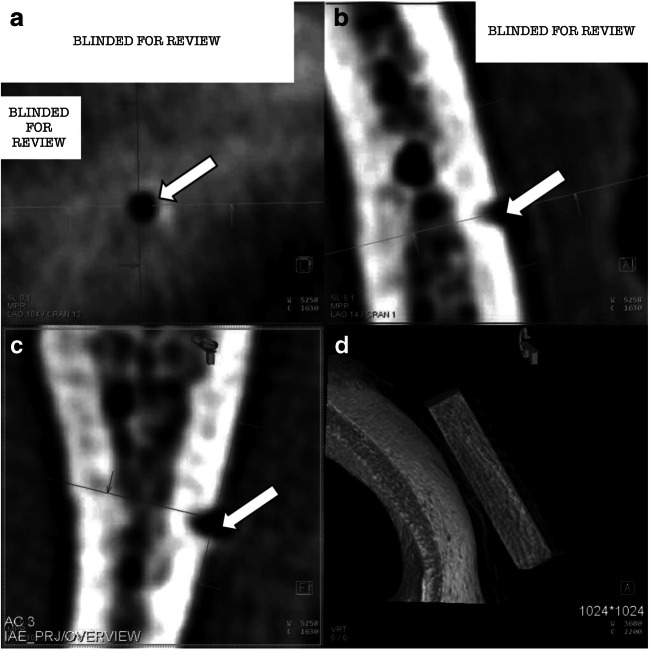
Artis Zeego workstation (Syngo XWP VD 11B) in the DICOM

Utilized equipment were a radiolucent HFD (DORO® Skull Clamp Radiolucent, pro med instruments GmbH, Freiburg, Germany), two types of titanium made pins (adult and child) (pro med instruments GmbH, Freiburg, Germany) (Fig. 2; schematic illustration including pin detail of the pins utilized in the experiment. Figure 2a shows the dimensions of the adult pin and Fig. 2b the pediatric pin. All dimensions are given in mm unless otherwise indicated.), a radiolucent reference scale (phantom) (Fig. 3; lateral view of the fresh-frozen skull obtained by Siemens Artis Zeego® in volume rendering showing the white 3D reconstruction box centered on the location of the pin. The white arrow shows the phantom placed next to the single pin.), and an angiographic CBCT (Artis Zeego, Siemens AG, Forchheim, Germany). The phantom made of bone analog material (short fiber-filled epoxy sheet, SAWBONES®, Vashon Island, USA) was designed for the study to monitor the accuracy of the CBCT datasets. Twenty-five holes of different depths, ranging from 0.11 to 2.0 mm, and diameters, ranging from 0.2 to 2.0 mm, allowed to estimate the margin of error measuring the puncture holes utilizing the measuring function of the Artis Zeego workstation (Syngo XWP VD 11B) in the DICOM (Fig. 4; selection of the hole of interest (white arrows) on a multiplanar reconstruction (MPR) to measure the size of the holes). The three orthogonal planes are oriented in order to be placed as the following: one tangential to the hole and at the surface of the skull (Fig. 4a) and the two others parallel to the axis of the hole (Fig. 4b and c). Figure 4d shows the multiplanar reconstruction. Some partial volume effect artifacts are visible in grayish around the hole visible in black.

### Experimental design

Three factors were considered to study the response of skull bones to pin fixation. The first factor was the pin-tip angle with two levels. 35°, utilized among adult population and 43° utilized among pediatric population. The pin-tip angles chosen for the experiment correspond to those used in clinical applications and commercially available. The second factor was the HFD clamping force. Forces utilized were 180, 270, and 360 N, induced with clamping screw located at the single-pin side of the HFD. Ninety N was excluded from the study because a feasibility study prior to the experiment showed no visible perforation of the bone. The third factor corresponded to the four different locations of the HFD given above. Table 2 links the bones pinned by the single pin of the HFD at the different approaches. To analyze the effect of these three factors on the depth of the pin-induced puncture holes, data were subjected to a three-way ANOVA with a subsequent post hoc test utilizing SPSS [7] as well as RStudio [5, 13, 15].

**Table 2:** Pinned cranial bone in the different approaches

Approach	Cranial bone pinned by the single pin	Cranial bone pinned by the pins of the two pin side
Bifrontal	Parietal: superior and posterior to the external auditory meatus (EAM^a^)	Parietal: superior and toward the midline Temporal: mastoid process
Pterional	Frontal: anterosuperior to the pterion	Occipital: superior and lateral to the occipital protuberance Temporal: mastoid process
Middle Fossa	Frontal: superolateral to the nasion	Occipital: superior and lateral to the occipital protuberance
Prone/ventral position	Parietal: superior to the EAM	Parietal: superior and posterior of the EAM, superior and anterior of the EAM

^a^External auditory meatus

### Description of the technique

Prior to any manipulation, a pre-operative CBCT covering the whole skull was performed for each specimen to exclude specimens with cranial bone pathologies or structural abnormalities.

A. *CBCT acquisition:* the specimen was pinned with the head holder (HFD) according to the parameters described above. The phantom was placed next to the single pin in order to be in the same volume of interest of the analyzed hole. For each pinning, two CBCTs were acquired: one with the HFD and a second after removal.
B. *Measurement of pin-induced puncture holes:* the puncture holes were assessed on the CBCT images without the HFD to visualize the puncture without strike artifacts induced by the radio opacity of the pins using the Artis Zeego workstation. For measurement accuracy purposes, we performed at the pinning location a resampled 3D reconstruction on the CBCT without HFD using a smaller volume of interest (VOI) and a sharp kernel algorithm to improve bone definition. To localize the reconstruction VOI on the CBCT image without HFD, the CBCT with the HFD was merged. The diameter was measured in the 3D reconstruction of the VOI. A direct measurement of the depth was not serviceable since the boundaries of the puncture holes in cross-section were blurry. Therefore, a formula (formula 1) was utilized to deduce the depth (*h*) as a function of the diameter (*d*) and known pin angle (*α*) as well as a constant (*c*) that differs between pediatric (*c* = 0.346) and adult pins (*c* = 0.470).
  1 $$ h=\frac{d\div 2}{\mathrm{tan}\upalpha}-c $$
C. *Analysis of the data:* the data obtained were checked for homoscedastic utilizing Levene’s test and then subjected to a 3-way ANOVA. Residuals of the 3-way ANOVA were then tested for normality utilizing Shapiro–Wilk test. Multiple comparisons via Tukey HSD test with *α* = 0.05 was conducted to analyze the differences between different treatments. Tukey HSD test was conducted within both pin-angle groups.

## Results

Each factor has a significant effect on the depth of the puncture (pin angle *p* < 0.001; pinning force *p* < 0.001; location *p* < 0.001). The interaction between location and force as well as between angle and force has a significant effect too (*p* = 0.002; *p* < 0.001). The interaction of the location and angle of the pin as well as the interaction of the three factors do not have a significant effect on the depth of the puncture with *p* = 0.486 and *p* = 0.056, respectively (Table 3).

**Table 3:** Three-way ANOVA results, *p* values below 0.05 indicate significance (printed in italics)

Group tested	df	Sum Sq	Mean Sq	*F* value	*p* value
Location	3	0.949	0.316	7.744	*<0.001*
Angle	1	7.093	7.093	173.575	*<0.001*
Force	2	9.802	4.901	119.934	*<0.001*
Location:angle	3	0.101	0.034	0.826	0.486
Location:force	6	1.055	0.176	4.305	*0.002*
Angle:force	2	1.329	0.665	16.267	*<0.001*
Location:angle:force	6	0.548	0.041	2.235	0.056
Residuals	48	1.961	0.041		

**Fig. 5:**
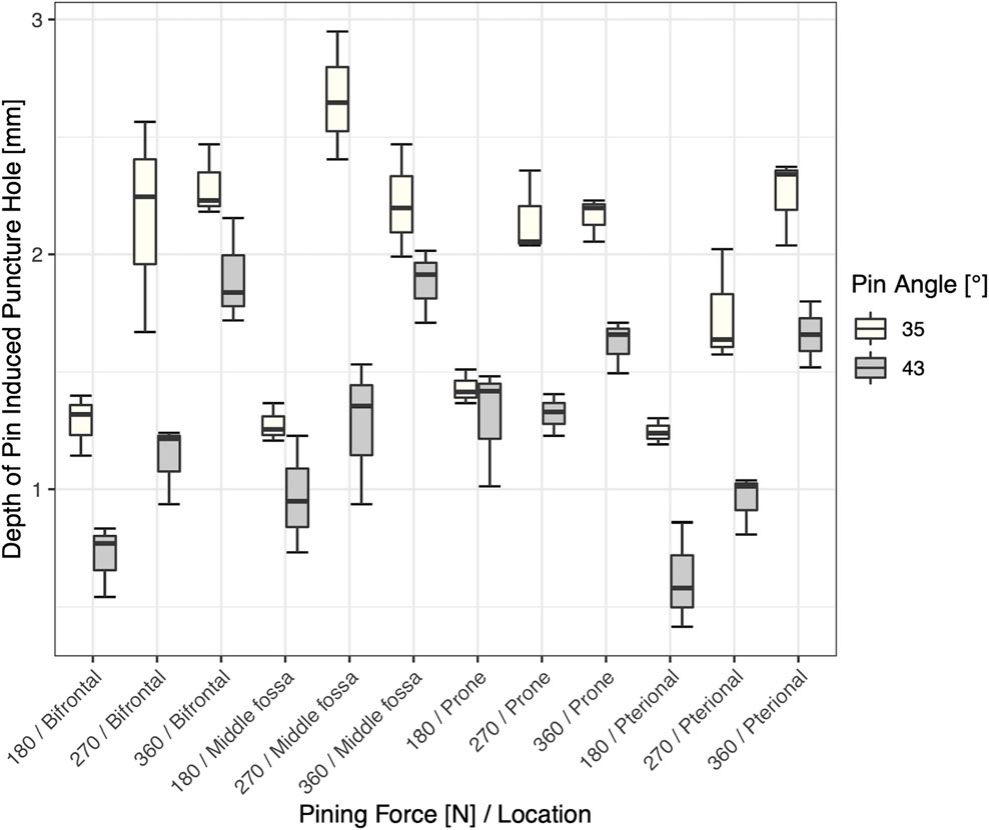
RStudio Team (2016). RStudio: Integrated Development for R. RStudio, Inc., Boston, MA, URL: http://www.rstudio.com

When applying the same pinning force and the same approach, the resulting punctures of pins with a pin angle of 43° are shallower than those induced with a pin angle of 35° (Fig. 5; boxplots of the depth of pin-induced puncture holes in the cranial bone as a function of pin angle, pinning force, and location (*n* = 3)).

**Fig. 6:**
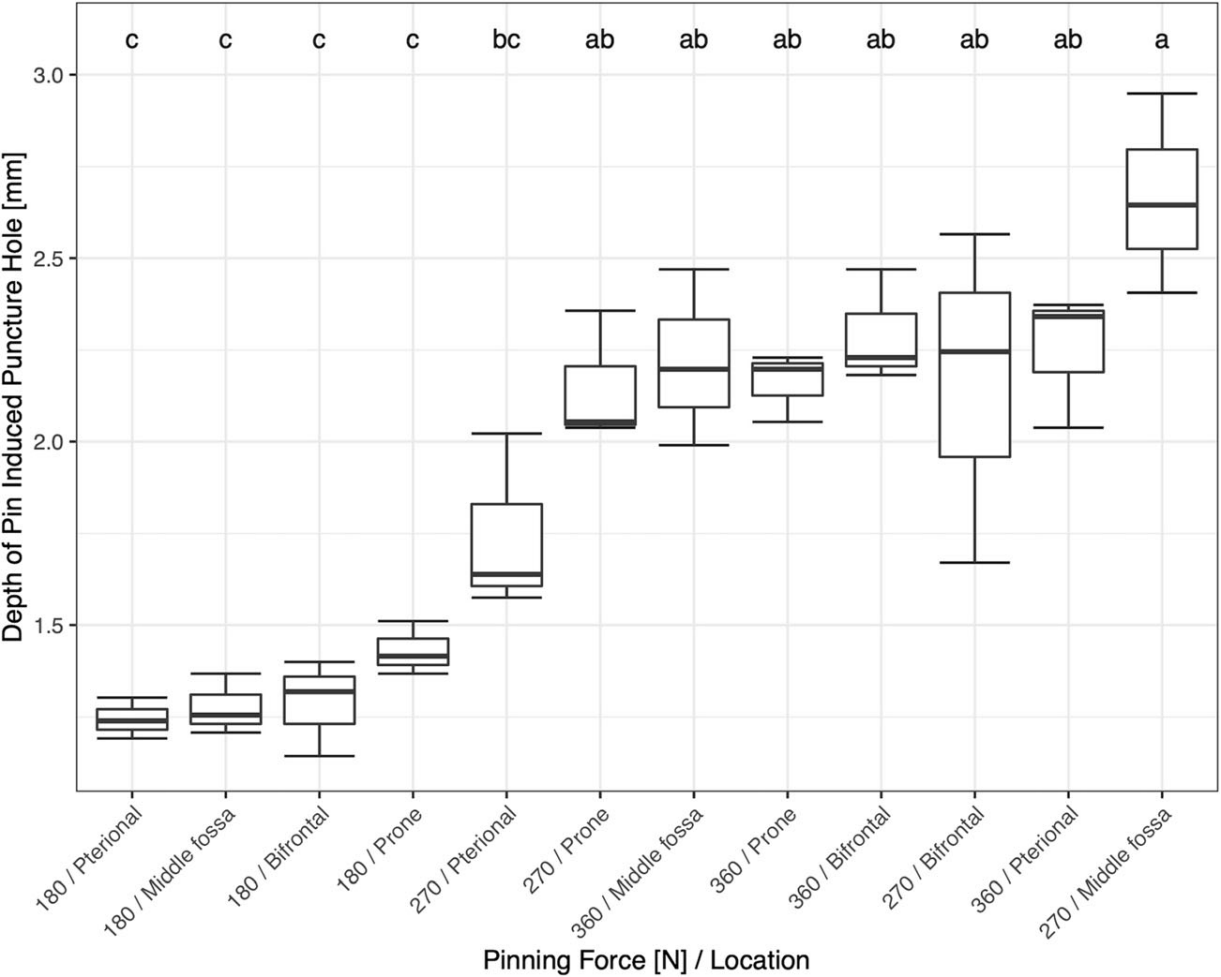
RStudio Team (2016). RStudio: Integrated Development for R. RStudio, Inc., Boston, MA, URL: http://www.rstudio.com

Among the pins with a pin angle of 35°, the deepest puncture, 2.67 ± 0.27 mm (mean ± SD), was recorded for a pinning force of 270 N in the middle fossa approach. This depth was not significantly different compared with puncture hole depths resulting from 270 or 360 N regardless of the approach except the depth recorded for 270 N and pterional approach (Fig. 6; boxplots of the depth of pin-induced puncture holes in the cranial bone of pins with a pin angle of 35° as a function of pinning force, and location (*n* = 3). Boxplots labeled with the same letters are not significantly different (*α* = 0.05) by Tukey HSD test.). The shallowest puncture, 1.25±0.06 mm, was recorded for a pinning force of 180 N in the pterional approach. All punctures resulting from 180 N belong to same significance class regardless of the tested approach (Fig. 6).

**Fig. 7:**
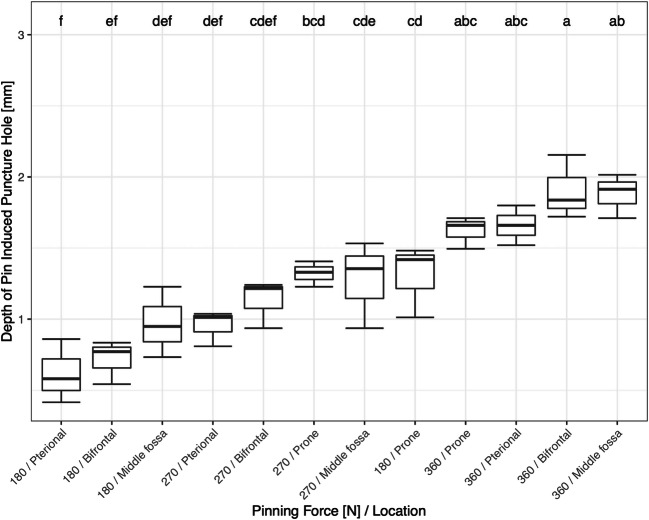
RStudio Team (2016). RStudio: Integrated Development for R. RStudio, Inc., Boston, MA, URL: http://www.rstudio.com

The deepest puncture of 1.90 ± 0.22 mm among the pins with the larger pin angle of 43° was recorded when pinning with 360 N in the bifrontal approach. However, this depth did not significantly differ from the recorded punctures induced by 360 N pinning force regardless of the approach (Fig. 7; boxplots of the depth of pin-induced puncture holes in the cranial bone of pins with a pin angle of 43° as a function of pinning force, and location (*n* = 3). Boxplots labeled with the same letters are not significantly different (*α* = 0.05) by Tukey HSD test). With 0.62 ± 0.22 mm in depth, the shallowest puncture was recorded when pinning with 180 N in the pterional approach. Compared with the other approaches at the same pinning force, the different puncture depths did not significantly differ except the prone approach. Moreover, the punctures recorded at 270 N in pterional and bifrontal approach, respectively, did not differ significantly from the three punctures with the smallest depth (Fig. 7).

## Discussion

The biomechanics of the interaction between the skull bones and the pins of the HFD is not yet a well understood despite its definite influence on complications. Despite the consequences, there is no standard method for using the HFD according to its modifiable parameters and the patient’s history. Increased clamping force and decreasing pin angle increase the observed depth of the puncture hole. In a previous experiment, Abdulhafez et al. [1] studied the biomechanics of the interaction between the pin and the bone and the main factors involved in complications. They demonstrated the influence of the clamping force on the degree of penetration of the pin and the occurrence of complications. The placement of the HFD is an interesting factor to discuss as its impact is unknown since it has not yet been studied.

The interaction between clamping force and location shows a significant effect on the depth of the puncture hole for a given pin angle. For the application of forces between 180 and 360 N as well as the 35° pin angle, the parietal bone pinned during the bifrontal approach and prone position has a similar reaction to the frontal bone in the superolateral part to the nasion used during the middle fossa craniotomy. For these approaches, a force of 270 N is sufficient to see a difference in puncture depth from 180 N. However, an increase to 360 N makes no difference. Abdulhafez et al. [1] reported that the skull bone gradually stiffens as the penetration of the pin is increased, due to the increased compression of the material that pushes back the pin. In addition, since the frictional force increases with pin penetration, it slows down the degree of pin penetration.

Therefore, avoiding tightening the HFD to forces greater than 270 N with an adult pin could reduce complications related to excessive force. Is this sufficient to prevent slipping? To assess this risk, a focused study of the effect of these parameters on complications is necessary.

In contrast to the locations mentioned above, the area of the frontal bone, which is located anterosuperior to the pterion, reacts differently. A force of 360 N is required to see a difference in penetration compared with 180 N. The application of 270 N is not enough to make the difference in penetration depth. However, the response of the anterosuperior part to the pterion is significantly different from the superolateral part at nasion at 270 N. The same bone pinned in two places with the adult pin has a different reaction at 270 N. The frontal bone pinned at 270 N is more brittle on the anterior part where the neurovascular supraorbital structures and frontal sinuses are located. The fact that the positioning of the HFD during the pterional approach is difficult, the recommended broaching by Ballock et al. [2] of the pins at 90° to the bone may not be perfect and therefore creates a source of error for this approach. This difference can also be explained by the fact that the two pins pinned opposite the single pin are placed on different locations during the pterional and middle fossa approach. The occipital bone pinned primarily at the middle fossa approach has been described as the thickest of the skull by Peterson and Dechow [12]. This thickness may allow better penetration of the single pin in the opposite direction.

The depth of the pediatric pin-induced puncture is the same for the bifrontal, pterional, and middle fossa approaches at clamping forces of 180 and 270 N. However, the application of 360 N results in a deeper hole than the lower forces. The pin-bone contact surface is larger for the pediatric pin; therefore, more force must be applied to counterbalance the higher frictional force to drive the pin into the bone. Abdulhafez et al. [1] showed that a larger contact surface area reduces the penetration of the pin into the bone. Since their experiment focused on the softer skull of children, their reported penetration measurements were greater because the bone structure.

The parietal bone pinned in the prone position responds identically to the application of the different forces tested. Despite the increase in force, the superior part of the EAM does not allow more penetration of the pediatric pin.

There is a different resistance at 180 N between the superior part to the EAM of the parietal bone and the posterosuperior part as well as the anterosuperior frontal bone to the pterion. This can be explained by a difference in bone density between these different parts. Peterson and Dechow [11] showed differences in the thickness of the outer table of the skull at several sites in the parietal bone. According to their experiment in 2003 [12], the thinnest location of the parietal bone is posterior to the EAM, which in our study corresponds to the bifrontal approach. We find an increased depth for the bifrontal approach compared with the prone position by tightening the HFD to 180 N.

Therefore, depending on the location and the angle chosen, a greater clamping force does not always imply more penetration. Despite conjectures, applying more force does not increase the penetration of the pin into the skull per se. We have shown that the skull bones have different reactions to the placement of the HFD, and it is important to continue research in this area.

Using specimens as well as equipment from the operating room, this study is as close as possible to the response of living bone and actual conditions. The application of the HFD is done by the same operator throughout the experiment in order to limit operator-dependent differences. The specimens used were defrosted in advance in order to reproduce the reaction of living bone. A few studies have investigated the effect of freezing on the elastic properties of bone. “There is a minimal effect of freezing human specimen on the elastic properties of their bones” [12].

Our study has limitations. Errors may occur when using different specimens to test different forces. The flexibility of the bone in each specimen may differ. Freezing and thawing have an effect on the biomechanical properties of the bone of the specimens. According to Unger et al. [17], fresh/frozen specimens remain the best choice for experiments involving the biomechanical properties of cortical bone. The specimens utilized were craniums without torso that may influence the penetration of the pin as gravity acceleration differ compared with the situation during clinical application. A potential effect of different scalp thickness on pin penetration depth was neglected. Utilized pinning forces were sufficient to puncture through the scalp of all specimens. In this state, the scalp was circular distended, and friction between the metal pin and the scalp is neglectable due to the lack of resilience of the skin tissue of the specimens. Since the CBCT workstation does not allow setting the same contrast for all datasets, measurement differences were identified when the contrast was changed. Furthermore, the spatial resolution, even high with a matrix of 512 × 512 (0.14 mm), induced some partial volume effect artifacts for the measurement of such small structures. This could also influence the measurement accuracy. In order to disclose this accuracy, we have calculated a measurement error to identify accuracy defects in the analysis of the DICOM. This measurement error compares the measured holes on CBCT and the reference phantom described above with known diameters. The measurement errors are greater for a small puncture hole diameter than a large one. The mean margin of error of the measurements was 22.1% for pins with 35° pin angle and 14.4% for pins with 43° pin angle. This difference was greater for the images with the adult pin compared with the child pin because the contrast was more difficult to adjust due to the fact that the composition of the pins might not be exactly the same.

Further investigation of the relationship of the tree factors to complications would allow specific recommendations to be made to neurosurgeons on optimal HFD placement based on patient history and planned approach. Subsequent studies could lead to a formula calculating the appropriate force to be applied based on the patient's cranial thickness. These studies could be used to develop a new generation of three-pin HFD that would reduce the risk of complications.

## Conclusion

To date, there is no standardized method and ideal clamping force for attaching a neurosurgical HFD. It is important to understand its impact on the skull in order to make evidence-based recommendations its utilization to reduce complications. Skull bones react differently to clamping force, and pin. We recommend that this type of research shall continue to help surgeons to reduce complications related to the placement of the HFD needed for surgery, adapt the fixation of the instrument for each patient, and to develop future HFDs.
